# Assessing Spatial and Spatiotemporal Tactile Working Memory Using Adaptive Staircase Procedures

**DOI:** 10.3390/s26082361

**Published:** 2026-04-11

**Authors:** Nashmin Yeganeh, Ivan Makarov, Runar Unnthorsson, Árni Kristjánsson

**Affiliations:** 1 The Faculty of Industrial Engineering, Mechanical Engineering, and Computer Science, University of Iceland, 102 Reykjavík, Iceland; 2 Icelandic Vision Laboratory, Faculty of Psychology, University of Iceland, 102 Reykjavík, Iceland

**Keywords:** vibrotactile wearable device, tactile feedback, stimuli discrimination, tactile working memory, vibrotactile patterns, haptic interface

## Abstract

Tactile working memory limits the amount of information that can be processed through touch, with important implications for the design of haptic communication systems. Although visual and auditory working memory have been extensively investigated, tactile working memory, particularly for spatial and spatiotemporal sequences, remains less well understood. The present study examined tactile working memory capacity in two psychophysical experiments. Participants reproduced sequential vibrotactile stimuli delivered to the forearm via a 3 × 3 array of voice-coil actuators by entering responses through keypresses. Both experiments employed an adaptive 3-up/1-down staircase procedure, in which sequence length was adjusted according to response accuracy, and thresholds were estimated from reversal points. In Experiment 1 (Ordered Recall), participants reproduced both the spatial locations and the temporal order of stimulation, yielding a memory capacity threshold of approximately four items. In Experiment 2 (Unordered Recall), participants recalled only the set of stimulated locations without regard to order, resulting in a higher threshold of approximately five items. These results demonstrate that incorporating temporal sequencing demands into spatial recall substantially increases cognitive load and reduces effective tactile memory capacity. The findings clarify fundamental limits of tactile working memory and provide practical guidance for the development of haptic interfaces, wearable feedback systems, and sensory substitution technologies that must balance information complexity with human cognitive constraints.

## 1. Introduction

Working memory enables the temporary storage and manipulation of information, yet its capacity is sharply constrained. In the visual [1,2] and auditory domains [3,4], psychophysical and behavioral research has consistently shown that only a small number of items can be maintained under controlled conditions before working memory performance declines markedly. This upper limit, sometimes referred to as the “magical number four” [1,5,6,7], has often been considered to be a core bottleneck in human cognition, and auditory investigations using psychoacoustic and adaptive methods have generally confirmed these constraints. Memory performance is constrained by both limited capacity and time-dependent forgetting. Working memory supports the temporary storage and manipulation of information, but can hold only a small amount of information at any given time. In contrast, the Ebbinghaus forgetting curve shows that memory retention declines as a function of time after learning, with rapid initial forgetting followed by a slower rate of decay in the absence of reinforcement of the learning [8,9]. Despite growing interest in the tactile modality for various purposes, the understanding of vibrotactile working memory remains substantially less developed than that of visual and auditory memory. This gap is particularly consequential given the central role of precise tactile information in vibrotactile Braille, sensory substitution, assistive technologies, and wearable haptic communication systems. In the absence of accurate, modality-specific estimates of spatial and spatiotemporal tactile working memory capacity, both theoretical frameworks and practical designs risk systematically exceeding the limits of human tactile information processing.

Staircase methods constitute a fundamental tool in psychophysics for quantifying perceptual and cognitive limits, yielding efficient and unbiased estimates of performance thresholds. The conceptual foundations of adaptive testing were introduced by Cornsweet (1962) and subsequently formalized by Wetherill and Levitt (1965 and 1971) through the transformed up–down staircase procedure [10,11,12]. Within this framework, task difficulty is systematically increased following correct responses and decreased after errors, promoting rapid and reliable convergence on an individual’s threshold level. As a consequence, staircase procedures enable robust, participant-specific threshold estimates. A central goal of staircase methods is the identification of reversal points, defined as trials at which the direction of difficulty adjustment changes—shifting upward following correct responses and downward following incorrect responses. Different staircase algorithms impose distinct response criteria for these reversals, requiring varying numbers of correct or incorrect responses to trigger a change in difficulty. For example, in a 1-up/2-down staircase, task difficulty decreases after each incorrect response but increases only after two consecutive correct responses. Thresholds are then estimated by averaging the stimulus levels at the reversal points towards the end. These reversals mark the transition zone between consistent success and failure and therefore tend to cluster tightly around the participant’s perceptual threshold. Averaging across reversal points yields a stable and unbiased estimate of sensitivity with substantially fewer trials than constant-stimulus methods, while simultaneously reducing the influence of random guessing and improving the reliability of threshold estimates [13,14].

Using this approach, the minimal vibrotactile force detectable by touch across different frequencies was measured using an adaptive staircase procedure. Results indicated that fingertip sensitivity peaks near 250 Hz, with thresholds around 1 mN, providing reliable psychophysical data for force-based vibrotactile perception [15]. It has also been shown that adding subthreshold vibrotactile stimulation can reduce the electrotactile perception threshold. Weak, imperceptible vibration was found to lower the voltage required for detecting electrical stimuli by approximately 3–5% [16]. Furthermore, adaptive staircase procedures have been demonstrated to produce lower and more consistent estimates of vibrotactile detection thresholds than traditional yes–no methods, suggesting that staircase-based psychophysical measurements provide more efficient and reliable threshold estimation [17].

Over the past several decades, adaptive psychophysical methods have been extensively refined to increase the efficiency and accuracy of threshold estimation in psychophysical research [17,18,19]. In auditory research, adaptive threshold-tracking techniques have been effective in refining tone detection and auditory discrimination paradigms, enabling rapid, reliable, and objective estimation of perceptual thresholds across a broad range of experimental contexts [20,21].

In perception research, task difficulty is often adjusted according to participants’ responses, allowing researchers and clinicians to assess visual sensitivity to contrast and to examine improvements in visual learning over time [19,22].

Adaptive threshold-tracking methods for vestibular motion perception have been evaluated using theoretical analysis, simulations, and whole-body motion experiments involving platform-induced linear and rotational movements designed to directly stimulate the vestibular system. These studies have demonstrated that optimized staircase algorithms yield reliable and efficient motion detection thresholds, substantially reducing the number of trials required for convergence [23].

Adaptive staircase procedures have been applied to estimate the minimum spatial separation required for reliable discrimination of mid-air vibrotactile stimuli. This approach yielded a gap-detection threshold of approximately 32 mm, providing quantitative evidence of the spatial limits of non-contact tactile perception [24]. Staircase methods have also been employed to characterize vibrotactile detection thresholds across a broad frequency range using a multichannel wearable interface equipped with 12 independently controlled actuators. Measurements obtained at six frequencies between 100 and 1000 Hz revealed highly consistent thresholds across individual fingers and between hands, with slightly elevated thresholds on the palm and peak sensitivity occurring near 250 Hz. These findings confirm the uniformity and reliability of the device for controlled psychophysical assessment of tactile sensitivity [25].

Additional work has investigated detection thresholds for ultrasonic travelling-wave vibrotactile stimuli, reporting minimal detectable amplitudes of approximately 1.5 µm [26]. More broadly, theoretical and simulation studies have examined the statistical properties of threshold-tracking procedures, showing that threshold and point-of-subjective-equality (PSE) estimates can be obtained with high accuracy when experimental parameters are appropriately configured [12,14,27].

Despite their simplicity, staircase methods provide robust and efficient psychophysical measurements across sensory modalities when properly parameterized. In this study, an adaptive staircase procedure was used to estimate vibrotactile detection thresholds.

The architecture of vibrotactile working memory remains poorly understood, particularly regarding the relative contributions of spatial and spatiotemporal recall in sequential vibrotactile stimulation and the limits on the number of items that can be retained. A particularly challenging issue for assessing tactile working memory is that different parts of the body have different tactile resolutions, complicating direct comparisons and threshold estimations across sites [28].

The research presented here forms part of a broader program aimed at developing a multi-purpose vibrotactile feedback device designed to be worn on the forearm. The device is intended to provide environmental feedback for individuals with visual or auditory impairments, as well as to assist users of prosthetic limbs by conveying proprioceptive information, for example, signals about joint position or movement, through tactile stimulation.

Spatial recall, or memory for which skin locations were activated, supports stable tactile patterns such as those used in Braille. Spatiotemporal recall—memory for both location and order—enables the encoding of sequences akin to language or music. Our current experiments were designed to compare performance across two tasks: remembering both the order and the location of vibrotactile stimuli versus remembering location alone. Our aim was to assess the additional memory costs of reporting the vibrotactile stimulation in the correct sequence while keeping spatial content constant. We hope that the results can provide practical guidance for haptic systems that need to balance accuracy, usability, and cognitive load.

Our group has extensively investigated vibrotactile perception over the past decade, with a particular focus on wearable array systems and their underlying design principles. This work builds upon recent studies that have systematically explored fine-tuned parameters and spatiotemporal encoding strategies. The current study incorporates these insights to deliver high-resolution stimuli, including finely tuned inter-actuator spacing and spatial placement to ensure distinguishable stimulation, as well as carefully designed vibratory features and temporal dynamics. Additionally, appropriate vibrotactile presentation methods are selected to enhance perception on the skin. Together, these design considerations aim to minimize sensory blending and enable high-resolution, distinct pattern perception on the skin [29,30,31,32,33]. Additional related work from our group on vibrotactile perception and haptic interfaces can be found on the Laboratory of ACUTE website at https://acute.hi.is (accessed on 5 April 2026). We have, in these studies, chosen the forearm as the stimulation site because it is relatively passive, does not interfere with daily activities, and allows for unobtrusive stimulation, making it particularly suitable for sensory substitution and assistive technology applications. Building on our previous evidence that sequential presentation leads to better tactile recognition than simultaneous stimulation [33], we assessed spatial and spatiotemporal tactile working memory using sequential vibrotactile patterns delivered to the forearm. We applied adaptive staircase methods to estimate individual thresholds for recalling vibrotactile sequences, using reversal points to estimate the thresholds for vibrotactile memory on the forearm.

The overarching aim of this study was to characterize the capacity limits of tactile working memory under differing recall demands using a pattern reproduction task. In Experiment 1 (Ordered Recall), participants were required to reproduce both the spatial locations of stimulated actuators and their temporal order. In Experiment 2 (Unordered Recall), participants recalled only the set of stimulated locations, irrespective of order.

Together, these experiments were designed to determine threshold sequence lengths for ordered (spatial + temporal) versus unordered (spatial-only) tactile working memory. Adaptive staircase procedures were employed to enable efficient and reliable estimation of these thresholds. Beyond their theoretical relevance, the findings are intended to inform the design of vibrotactile communication systems and sensory-substitution technologies by providing guidance for more effective strategies for conveying information through touch.

## 2. Materials and Methods

### 2.1. Participants

Ten healthy adults (seven men and three women), aged 26–40 years (M = 33.6, SD = 5.6), participated in the study. All participants were right-handed and reported normal tactile sensitivity, with no known history of neurological or sensory disorders that could affect somatosensory perception. Participant eligibility was determined by self-report screening prior to enrolment. All experimental procedures were conducted in accordance with the ethical principles outlined in the Declaration of Helsinki, and informed consent was obtained from all participants prior to participation.

### 2.2. Stimuli and Vibrotactile Wearable

Vibrotactile stimulation was delivered using voice-coil actuators (VCAs) (Lofelt L5; Lofelt, Berlin, Germany). These actuators were selected for their fast response times, high force output, and low power consumption (Table 1), making them well-suited for wearable vibrotactile applications. Their compact form and independent control capabilities allow reliable integration into multichannel wearable systems. Designed to deliver rich vibrotactile feedback, these actuators enable independent control of both frequency and amplitude, as detailed in Table 1.

The L5 actuators measure W: 17.0 mm × D: 20.5 mm × H: 6.2 mm in their static state and expand to W: 17.0 mm × D: 25.5 mm × H: 6.2 mm when vibrating at maximum displacement (though they may not always operate at this level). To accommodate this motion, custom 3D-printed enclosures were designed with slightly larger dimensions (18.0 mm × 29.0 mm × 7.2 mm), and the wall thickness was 0.5 mm.

**Figure 1:**
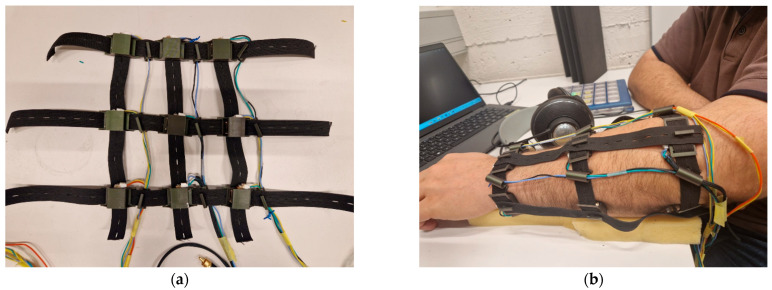
The wearable vibrotactile sleeve used in the study: (**a**) illustrates the inner side of the sleeve (that makes contact with the skin), featuring nine actuators arranged in a 3 × 3 grid around the forearm; (**b**) shows the sleeve worn on the user’s arm, displaying the outer side with a custom strap system that secures the actuators in place. The design ensures an even distribution of vibrotactile stimulation and allows for adjustable positioning across different forearm sizes.

A wearable vibrotactile system with nine actuators arranged in a 3 × 3 grid around the forearm was custom-developed for the experiment (see Figure 1).

This configuration was designed to ensure an even distribution of tactile feedback across both the top and lateral surfaces of the forearm, enabling immersive and effective haptic experiences. The grid layout provides precise spatial control of the stimulation; each actuator was securely mounted within its enclosure, ensuring stability while also allowing free vibration along the primary axis of motion, creating the vibration. This design enabled the actuators to operate as a unified vibrotactile system, minimizing mechanical damping and preserving the fidelity of the haptic output.

To securely mount vibrotactile actuators on the forearm, a custom stretch-strap system was developed. Each strap is made from spandex—a soft, elastic material that naturally conforms to the shape of the user’s arm. This stretchable fabric ensures a snug, comfortable fit to the arm while maintaining consistent skin contact. In short, the grid configuration enables accurate and intuitive vibrotactile feedback.

The straps feature pre-punched holes that allow for adjustable positioning, accommodating various forearm sizes. These holes interface with a small rear-mounted pin on each enclosure, securely anchoring the actuators in place. Additionally, each enclosure includes integrated side slots that the straps pass through, allowing multiple actuators to be mounted along a single strap and wrapped around the forearm in a stable configuration. This setup enables users to easily adjust actuator placement to match their forearm size and ensures even distribution of the actuators around the forearm. Velcro fasteners were used to secure the strap system in place. This strap-based approach offers significant advantages, especially for prototyping and user-testing.

This setup ensures that the actuators can be repositioned or removed quickly without disassembling the entire setup, making the system ideal for iterative design. Its ability to adapt to a wide range of forearm shapes and sizes eliminates the need for rigid, one-size-fits-all components, supporting an inclusive and user-centered design.

### 2.3. Wearable Equipment for Stimulus Presentation

**Figure 2:**
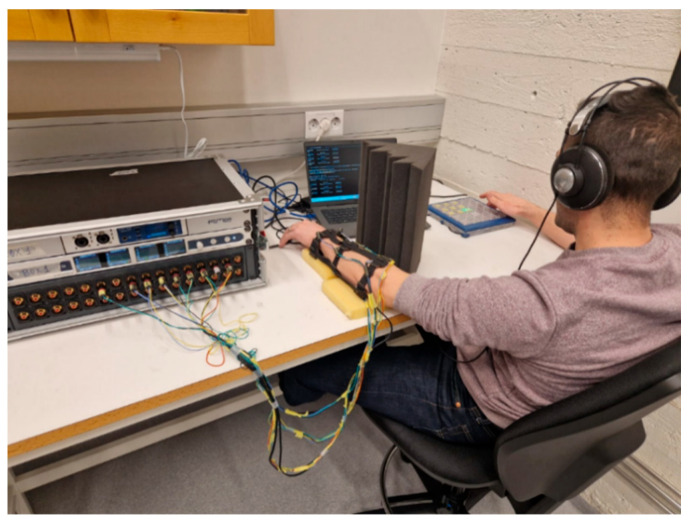
Experimental setup and hardware configuration. A participant wears a vibrotactile sleeve while resting the forearm on a padded support. White noise is delivered through over-ear headphones to mask actuator sounds. Participants respond using a keypad to reproduce the stimulation sequence.

The system for stimulus presentation involved a digital audio interface (RME MADIface XT; RME Audio, Haimhausen, Germany), digital-to-analog converters (Ferrofish A32; Ferrofish, Linz am Rhein, Germany), multi-channel amplifiers, and Lofelt L5 voice coil actuators. The tactile sleeve was connected to the audio hardware with wired connections, allowing precise control of the vibrotactile output. Vibratory features were controlled using Python (version 3.9) code, which generated digital signals. These signals were transmitted via USB to the audio interface and then routed to digital-to-analog converters. The resulting analog signals were amplified and sent to the voice coil actuators, producing the desired vibrotactile stimuli (the experimental setup is presented in Figure 2).

### 2.4. Procedure

#### 2.4.1. Participant Preparation and Sensory Isolation

Participants wore custom-designed wearables incorporating nine vibrotactile actuators distributed evenly around the forearm. During testing, the stimulated hand rested on a padded support to minimize fatigue and involuntary movement. Visual access to the stimulation site was blocked using a padded occluding shield to eliminate visual cues. To prevent auditory information from influencing performance, participants wore over-ear headphones that continuously delivered white noise throughout the experiment, most importantly, masking any audible cues generated by the actuators but also any environmental sounds (Figure 2).

#### 2.4.2. Training and Familiarization Phase

Before data collection, participants completed a structured training session to familiarize them with the hardware setup, vibrotactile stimuli, interstimulus timing, and response procedure. During this phase, participants practiced identifying and reproducing both single-actuator and multi-actuator stimulation patterns. Corrective feedback was provided throughout the training to ensure accurate understanding of the spatial layout, actuator mapping, and task instructions. This training phase ensured that all participants clearly understood the task requirements and were fully familiar with the experimental procedure before the main trials began. No feedback was provided during the experimental trials to avoid learning effects and to ensure that performance reflected stable tactile working memory processes.

#### 2.4.3. Experimental Task and Adaptive Staircase Procedure

The study comprised two experiments designed to estimate tactile sequence memory thresholds using adaptive staircase procedures. Both experiments employed identical apparatus, stimulus generation, and staircase parameters; the sole difference lay in the reporting requirement. In the ordered recall condition, participants were required to reproduce both the spatial locations and the temporal order of the vibrotactile stimuli, whereas in the unordered recall condition, they reported only the set of stimulated locations, irrespective of order. Each experiment lasted approximately 40 min.

The two experiments were conducted on separate days with several days between sessions to reduce potential short-term practice (28 days). Participants were not informed about the second experiment during the first session, preventing them from adapting their encoding strategies in anticipation of a different recall requirement.

The study employed a within-subject psychophysical design in which all participants completed both experimental conditions and contributed multiple repeated observations. Such designs are commonly used in controlled psychophysical research because each participant serves as their own control, reducing between-participant variability and increasing sensitivity to systematic differences between conditions.

Vibrotactile stimuli were presented as sequences of discrete pulses delivered to distinct actuator locations, with each actuator activated no more than once within a given sequence. Sequence length was adjusted adaptively according to a 3-up/1-down rule: following three consecutive correct responses, the sequence length increased by one item, whereas a single incorrect response resulted in a decrease of one item.

The 3-up/1-down staircase procedure has been shown to converge on a performance level of approximately 79% correct, providing a robust and unbiased estimate of the threshold on the psychometric function [12,13]. Each staircase continued until six reversal points were obtained, and the threshold for each participant was defined as the mean sequence length across these reversals. Using six reversals yields a reliable and stable threshold estimate, consistent with psychophysical recommendations indicating that six to eight reversals are sufficient for accurate convergence [12].

Although the apparatus, stimulus generation, and staircase parameters were identical across experiments, the recall requirements differed fundamentally between the two conditions. In Experiment 1 (Ordered Recall), conducted on a single day, participants were required to reproduce both the spatial locations of the stimulated actuators and their temporal order of activation. Responses were entered sequentially on a keypad to match the order of stimulation. For example, a sequence presented as A → C → B was considered correct only if reproduced exactly as “A–C–B.” Responses were scored as incorrect if an incorrect actuator was reported or if the correct actuators were entered in the wrong order.

In Experiment 2 (Unordered Recall), conducted on a separate day after completion of Experiment 1, participants completed an otherwise identical procedure but were required to recall only which actuator locations had been stimulated, irrespective of order. Using the same example sequence (A → C → B), any response containing the set {A, B, C} was scored as correct. Errors were recorded only if one or more stimulated locations were omitted or if an incorrect location was reported.

Each vibrotactile pulse was presented for 500 ms, separated by a 450 ms interstimulus interval (ISI), at a stimulation frequency of 100 Hz and an intensity of 9.6 g. These parameters were selected based on prior work demonstrating their suitability for sequential vibrotactile perception tasks [30,31,33].

## 3. Results

**Figure 3:**
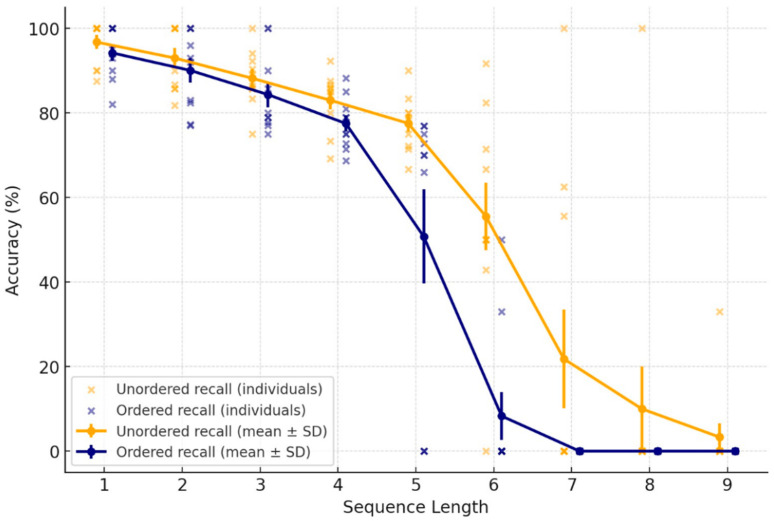
Accuracy trends across patterns. Mean accuracy (%) for Ordered (blue) and Unordered (orange) recall across sequence lengths. Error bars indicate variability across participants (±SD). Accuracy declined more steeply for Ordered recall beginning at Pattern 5, and for Unordered recall beginning at Pattern 6. Crosses along the x-axis mark sequence lengths not attempted by some participants due to the termination of their adaptive staircases after six reversal points at shorter lengths.

All participants completed both experiments—ordered recall (Experiment 1) and unordered recall (Experiment 2)—on two separate days. Because the same individuals took part in both, this allowed us to use recall type as a within-subjects factor, enabling direct comparison using repeated-measures ANOVA. A two-way repeated-measures ANOVA was then conducted with recall type (ordered vs. unordered) and pattern length (number of vibrotactile stimuli per sequence) as factors, allowing us to assess their main effects on recall accuracy, as well as their interaction. Figure 3 shows the accuracy on the vibrotactile memorization task for Experiments 1 and 2, as a function of the length of the sequence to be memorized.

The ANOVA analyses revealed a significant main effect of recall Type, F (1, 9) = 18.96, *p* = 0.002, η^2^_p_ = 0.678, demonstrating that accuracy differed significantly between the two experiments. Accuracy was consistently higher for unordered recall (Experiment 2), indicating that removing the demand to reproduce the stimulation sequence in the order of presentation led to higher accuracy, presumably because this involved lesser cognitive load.

A large main effect of Pattern Length was also observed, F (8, 72) = 116.96, *p* < 0.001, η^2^_p_ = 0.929. Across both experiments, accuracy decreased as the number of stimuli in a sequence increased, reflecting the capacity limits of tactile working memory. There was also a significant interaction between recall type and pattern length, F (8, 72) = 8.06, *p* < 0.001, η^2^_p_ = 0.472, reflecting that the decline in accuracy with increasing sequence length was steeper in experiment 1 (ordered recall), where participants had to encode and report both spatial locations and temporal order.

### 3.1. The Effect of Pattern Length and Recall Type on Tactile Recall

In Experiment 1 (Ordered Recall), participants were required to reproduce both the spatial locations of the stimulated actuators and their temporal order (e.g., a sequence A → C → B had to be recalled exactly as “A–C–B”). In Experiment 2 (Unordered Recall), participants reported only the set of stimulated locations irrespective of order, such that any response containing {A, B, C} was scored as correct for the same sequence. Figure 3 presents mean recall accuracy across nine vibrotactile sequence lengths for both conditions, with accuracy (percentage correct) plotted as a function of pattern length (1–9 items).

As sequence length increased, accuracy declined in both conditions, indicating progressively greater demands on tactile working memory. For short sequences (Patterns 1–3), performance was near ceiling in both experiments. Accuracy ranged from 84.66% to 93.92% for ordered recall and from 88.18% to 96.75% for unordered recall, demonstrating that sequences of up to three items could be recalled with high accuracy regardless of recall type. Beyond this range, performance diverged systematically between conditions. In the ordered recall condition, accuracy declined for Pattern 4 (≈79%) and decreased sharply for Pattern 5 (≈51%), reaching near-floor levels for sequences of six items or more (≈9–0%). In contrast, in the unordered recall condition, accuracy remained relatively high for Pattern 5 (≈77%), declined more gradually for Pattern 6 (≈62%), and continued to decrease across Patterns 7–9 (≈22%, 10%, and 3.3%).

The error bars in Figure 3 (±SD) further characterize these effects. At longer sequence lengths, variability was substantially greater in the unordered recall condition, reflecting considerable interindividual differences in spatial tactile working memory capacity. In contrast, variability in the ordered recall condition was comparatively low, with performance declining more uniformly across participants, consistent with a general constraint on the ability to encode and retain temporal order information.

To determine the sequence lengths at which performance changes became statistically reliable, post hoc analyses were conducted using Bonferroni-corrected comparisons. In the ordered recall condition, no significant differences were observed among Patterns 1–4 (all *p* > 0.05), indicating statistically stable performance across these sequence lengths despite a modest numerical reduction at Pattern 4. From Pattern 5 onward, however, accuracy declined significantly relative to Patterns 1–4 (*p* values ranging from 0.011 to 0.038). In the unordered recall condition, performance remained statistically stable up to Pattern 5, whereas significant declines emerged from Pattern 6 onward, with large reductions relative to earlier patterns (all *p* < 0.001).

Taken together, and as supported by the post hoc analyses and the significant Recall Type × Pattern Length interaction observed in the ANOVA, these results indicate that ordered recall can be reliably maintained for sequences of up to four items, whereas unordered recall extends to approximately five items before statistically significant performance deterioration occurs. Although temporal sequencing cannot be fully dissociated from spatial encoding in the present task, the statistical pattern of results demonstrates that the addition of temporal order requirements imposes a substantial and measurable increase in cognitive load relative to spatial-only recall.

### 3.2. Tactile Memory Thresholds

**Figure 4:**
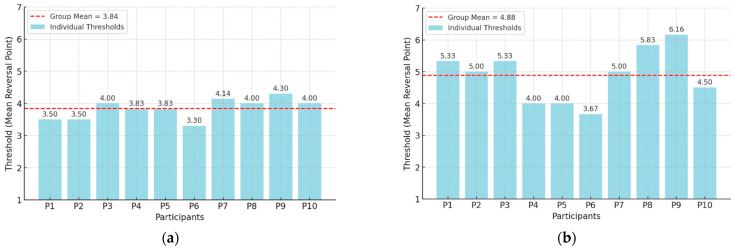
(**a**) Individual and Group Thresholds in the Memory Staircase Task (Ordered Response, Experiment 1); (**b**) Individual and Group Thresholds in the Memory Staircase Task (Unordered Response, Experiment 2).

Individual thresholds were defined as the mean sequence length across six reversal points. Figure 4 presents individual threshold estimates (Participants P1–P10) for both experiments, with the group means indicated by a red dashed line. Shapiro–Wilk tests confirmed that threshold distributions did not deviate significantly from normality in either condition (all p’s > 0.05), justifying the use of parametric summary statistics (means and standard deviations) for group-level reporting and comparison.

At the group level, thresholds were higher in the unordered recall condition (Experiment 2; M = 4.88, SD = 0.8) than in the ordered recall condition (Experiment 1; M = 3.84, SD = 0.3), representing an average increase of approximately one item when temporal order constraints were removed. The relatively small variability observed in the ordered recall condition likely reflects the stricter task requirements, as participants were required to encode both the spatial location and the temporal order of the vibrotactile stimuli. This constraint limits the range of possible encoding strategies and may lead to more uniform performance across participants. In contrast, the unordered recall condition allows participants to rely on alternative strategies such as spatial grouping or configuration-based encoding of the stimulation pattern. Differences in how participants apply these strategies may explain the somewhat larger variability observed in this condition. Notably, although all participants demonstrated improved performance in the unordered condition, the magnitude of this improvement varied between individuals, with some participants showing particularly large gains. Nevertheless, the advantage of the unordered recall condition was consistent across all participants, indicating that the removal of temporal order constraints systematically improves tactile memory performance.

These findings suggest that when recall is unconstrained by serial order, participants are able to adopt encoding and retrieval strategies that are unavailable or less effective under ordered recall demands. In contrast, ordered recall requires the concurrent maintenance of both spatial location information and temporal position, thereby placing additional demands on executive control and sequence monitoring processes. Although the present design does not permit a complete dissociation of temporal sequencing demands from spatial encoding alone, the results unambiguously demonstrate that enforcing correct temporal order substantially reduces the number of vibrotactile stimuli that can be retained in working memory.

This distinction has direct implications for the assessment of tactile working memory and for the design of vibrotactile communication systems. The observed individual threshold differences provide important insights into how task demands shape tactile memory capacity. When participants were required to remember only which spatial locations were stimulated, without preserving temporal order, they were able to reliably retain approximately five items. This higher capacity supports applications in which spatial information alone is sufficient, allowing greater information density to be conveyed through vibrotactile stimulation.

In contrast, when recall required both spatial location and temporal order, working memory capacity was reduced to approximately four items. This reduction reflects the additional cognitive demands imposed by maintaining serial order information alongside spatial representations. Critically, these results demonstrate that task requirements do not merely modulate task difficulty but fundamentally alter the amount of information that can be retained in tactile working memory. From an applied perspective, this finding underscores the importance of carefully selecting ordered versus unordered presentation schemes in haptic system design, as this choice directly constrains the volume of information that can be communicated reliably through vibrotactile interfaces.

## 4. Discussion

We examined the capacity limits of tactile working memory using vibrotactile stimulation and an adaptive staircase procedure. By comparing ordered recall (spatial location + temporal order) with unordered recall (spatial location only), we evaluated the additional cognitive load incurred when participants were required to encode and reproduce not only the spatial locations of stimuli but also their sequential order.

The results show that when observers need to remember the locations of vibrotactile stimulation and the temporal order that they were presented in, this results in significant reductions in accuracy, negatively affecting the memory thresholds for stimulus locations, relative to when recall of their temporal order is not needed. For all participants, the maximum number of remembered stimulation sites was higher for unordered than ordered recall, with an average increase in the estimated threshold for correct performance of just over one item. The increased difficulty observed in the ordered condition likely reflects the additional requirement to encode, maintain, and reproduce the temporal sequence of tactile stimuli. This task requires participants to simultaneously represent both the spatial locations of stimulation and their temporal order, thereby increasing the cognitive demands of the task. Maintaining these dual representations likely increases attentional load and the potential for interference between successive tactile stimuli. In addition, ordered recall requires participants to maintain a stable serial representation of tactile events in working memory, which further increases task complexity.

The interaction between recall type and sequence length highlights how ordered recall is more limited in capacity, with high accuracy maintained for only up to four items before performance declines sharply. Capacity is higher for unordered recall, however, supporting accurate performance for up to five items before any deterioration in performance occurs. Using spatial information alone allows a broader capacity buffer. This has important implications for information conveyance with vibrotactile displays.

It is important to relate our current results to a previous study where we tested the performance of simultaneous versus sequential presentation of Braille-like vibrotactile patterns [33]. There, we found a very large advantage for sequential presentation. Coupled with the current results, this suggests that sequential presentation, where the order of stimulated sites is unimportant, is a very efficient way of conveying information with vibrotactile displays on the forearm. This pattern is consistent with working memory models that distinguish spatial and temporal subsystems, such as Baddeley’s framework [35]. Spatial recall relies on processes similar to the visuospatial sketchpad, while spatiotemporal recall may depend on sequencing mechanisms like the phonological loop, which explains why adding temporal demands leads to worse memory performance.

Importantly, however, our design cannot establish whether temporal sequencing alone is more demanding than spatial encoding, since the ordered condition required participants to recall both spatial location and temporal order, but this was, in any case, not the aim of our study.

Informal reports from participants, obtained through a brief questionnaire administered after the experiment, provided additional insight into the strategies used during the task. When the stimulation order did not need to be reported, many participants described perceiving the vibrotactile sequence as a holistic “shape,” similar to visualizing a geometric figure. This suggests that participants relied on spatial grouping rather than sequential encoding, thereby reducing the effective memory load. Questionnaire responses also indicated that participants were generally able to clearly perceive and discriminate the vibrotactile stimuli. However, in the ordered recall condition, many reported difficulties maintaining the correct temporal sequence as pattern length increased. This suggests that errors in the ordered recall condition were primarily related to limitations in working memory rather than to perceptual difficulties in identifying the stimulation locations.

The strategy closely parallels findings in Braille reading, where dot configurations are perceived as integrated tactile patterns instead of being processed element by element. Such evidence highlights that spatial mapping can work as a natural cognitive resource [36]. Designing haptic systems that exploit this capacity—for example, by presenting information in spatially coherent rather than temporally fragmented forms—may improve usability, minimize cognitive demands, and support faster, more intuitive pattern recognition and recall.

This highlights the advantages of sequential spatial encoding for haptic and assistive technologies. Sensory substitution devices and wearable communication systems should therefore emphasize spatial distinctiveness while minimizing sequencing demands to enable recall of longer item sets, especially for symbolic or linguistic content where stimulation order is not critical. However, in situations where sequence order is essential, designers should consider the memory limits for ordered recall seen here to prevent cognitive overload and performance decline.

Exceeding these thresholds risks sharp declines in accuracy, which could compromise communication efficiency and users’ trust in the information conveyed by the device. In practical terms, vibrotactile communication on the forearm is more efficient when information is encoded spatially rather than as ordered sequences. Requiring users to remember stimulus order reduces the maximum usable sequence length from about five stimuli to about four. This highlights a clear trade-off between sequencing demands and information capacity that should be considered when designing wearable haptic systems.

Importantly, the present findings should be interpreted within the perceptual conditions of the current experiment. In previous work, we experimentally determined the minimum actuator spacing required to achieve reliable spatial discrimination on the forearm [32]. Based on those findings, the actuator spacing used in the present study was intentionally selected to exceed this resolution minimum, ensuring accurate identification of stimulation locations. In addition, the temporal parameters and vibrotactile stimulus characteristics were chosen within ranges where the stimuli were clearly perceivable and reliably discriminable [37]. Under these conditions, the observed capacity limits of approximately four to five items likely reflect tactile working memory performance when perceptual discrimination is relatively reliable. If actuator spacing were reduced toward the limits of spatial resolution, perceptual uncertainty in identifying stimulation locations would likely increase, which could in turn reduce the number of items that can be effectively encoded and maintained in working memory. Moreover, tactile acuity varies substantially across body sites due to differences in mechanoreceptor density. Consequently, stimulation delivered to lower-acuity regions, such as the forearm or back, may yield lower effective memory capacity compared to stimulation applied to more sensitive areas, such as the fingertips.

The present study examined capacity limits in tactile working memory rather than time-dependent forgetting. Nevertheless, the observed decline in recall accuracy with increasing sequence length can be considered in relation to classical descriptions of memory decline, such as the forgetting curve described by Hermann Ebbinghaus [8]. This comparison is useful because both phenomena produce a similar pattern of decreasing performance, even though they arise from fundamentally different mechanisms.

In Ebbinghaus’ experiments, memory was tested using nonsense syllables to minimize semantic associations, and retention was measured at progressively longer time intervals while the amount of information remained constant. These studies showed that forgetting follows a negatively accelerated function, characterized by rapid initial forgetting followed by a slower rate of decay.

In contrast, the present study manipulated memory load rather than time. The retention interval remained constant while participants were required to reproduce progressively longer vibrotactile sequences. Consequently, the decline in performance observed here reflects capacity limits of tactile working memory under increasing information load rather than the temporal decay of stored information.

Beyond classical accounts of memory decline, the present findings can also be interpreted in relation to previous research on tactile perception. Gallace et al. [38] describe the body surface as a communication channel with limited capacity for processing multiple tactile inputs. The thresholds observed here—approximately four items for ordered recall and five for unordered recall—fall within a comparable range. The higher capacity in the unordered condition may reflect spatial grouping strategies, whereby participants encode the overall “shape” of the stimulation pattern rather than processing each stimulus individually. Sensory interactions between successive stimuli may also influence performance. Cholewiak and Craig [39] showed that closely spaced vibrotactile stimuli can produce spatial and temporal masking effects. In the present study, the inter-stimulus interval (ISI) of 450 ms was chosen to reduce such interference and allow reliable identification of stimulation locations. A short ISI would likely increase sensory overlap and masking, which could impair discrimination and reduce the effective memory threshold for vibrotactile sequences.

In addition to the theoretical implications discussed above, several methodological considerations should be addressed when interpreting the present results. One methodological consideration concerns the fixed session order in which the two recall conditions were tested. The ordered recall experiment was conducted before the unordered recall experiment, which raises the possibility that performance differences between conditions could partly reflect practice or familiarity effects. Several aspects of the experimental procedure were implemented to minimize such influences. The two experiments were conducted on separate days with sufficient intervals between sessions (28 days) rather than within a single continuous session, thereby reducing short-term learning or fatigue effects. In addition, participants completed a training and familiarization phase before the experimental trials, allowing them to become comfortable with the vibrotactile sleeve and response procedure before data collection. Participants were also not informed about the second experiment during the first session, which reduced the likelihood that they would adjust their encoding strategies in anticipation of a different recall requirement. Although Familiarity effects cannot be completely excluded, the consistent performance advantage observed in the unordered recall condition—together with participant reports indicating the use of spatial grouping strategies when temporal order was not required—suggests that the observed difference primarily reflects the additional working memory demands associated with maintaining temporal sequence information. Nevertheless, the influence of order effects cannot be fully ruled out.

The present findings should also be interpreted within the context of the specific experimental design and stimulation configuration used in this study. In particular, the recall requirement—ordered versus unordered reproduction—was found to significantly influence the accuracy of vibrotactile sequence recall. This suggests that the operational definition of recall can substantially affect measured performance limits in tactile memory tasks. Consequently, estimates of tactile working memory capacity derived from vibrotactile experiments should be interpreted in relation to the specific task structure, stimulated body site, recall rules, and stimulation configuration employed in the experiment.

More broadly, performance in the vibrotactile recall task likely reflects the combined influence of several interacting factors, including tactile discriminability, spatial localization ability, attentional processes, encoding strategies, and response mapping, in addition to memory itself. Accordingly, the thresholds reported here should be considered estimates of performance limits within this specific forearm-based vibrotactile task rather than a universal measure of tactile working memory capacity. Because tactile acuity varies substantially across body regions and device configurations, the observed thresholds may depend on characteristics of the stimulation setup, such as the body site stimulated, actuator configuration, and vibrotactile stimulus parameters.

Despite these task- and device-specific constraints, the present findings provide preliminary insights into how sequence length and recall structure influence vibrotactile information processing. Given the exploratory nature of this study and the controlled experimental conditions under which the data were collected, the results should be interpreted as initial evidence rather than definitive estimates of tactile working memory capacity. Nevertheless, they offer useful guidance for the design of wearable haptic interfaces and vibrotactile communication systems.

In particular, designers should carefully consider the limits of tactile working memory when determining the amount and structure of information conveyed through vibrotactile stimuli. Encoding strategies that minimize sequencing demands and emphasize spatial distinctiveness may improve usability and information retention. More broadly, effective vibrotactile communication systems should balance information density with human cognitive constraints to ensure reliable perception, accurate recall, and efficient interaction.

Note that the sample comprised ten participants, which may appear modest; however, several methodological factors strengthen the reliability of the results. The observed effect sizes were substantial (η^2^_p_ = 0.678 for recall type and η^2^_p_ = 0.929 for pattern length), and the within-subject design increased statistical sensitivity while reducing interindividual variability. In addition, the adaptive staircase procedure produced stable threshold estimates across multiple reversal points, and all participants showed the same directional pattern, with higher thresholds in the unordered recall condition. A sensitivity analysis conducted using G*Power (version 3.1) further indicated that, with *N* = 10 in a within-subject design, the study was primarily powered to detect relatively large within-participant effects. Moreover, both the objective performance patterns and participants’ subjective reports were consistent with this interpretation, as participants indicated that stimulation locations were generally easy to perceive while the primary difficulty arose from maintaining the correct temporal order as sequence length increased. Nevertheless, the results should be interpreted as preliminary estimates obtained within the specific experimental paradigm used here.

Second, vibrotactile stimulation was applied exclusively to the forearm under controlled laboratory conditions. Although the forearm is a practical site for wearable applications, it is less sensitive than regions such as the fingertips [28]. Moreover, laboratory testing minimizes factors such as body motion, environmental distractions, and multisensory interference that are likely to be present in real-world use. Consequently, the memory thresholds reported here may differ for other body locations or in more ecologically valid contexts. Future research should therefore assess tactile working memory across multiple skin sites and under realistic usage conditions, including multisensory environments, for example, by examining interactions between tactile, visual, and auditory cues [40] to determine how these factors influence capacity limits and sequencing costs in applied settings.

Finally, the study focused specifically on spatial and spatiotemporal aspects of tactile working memory. Other stimulus attributes—such as vibrotactile intensity, duration, or frequency—were not examined. The reported thresholds should therefore be interpreted as estimates of spatial and sequence-based tactile memory rather than a comprehensive measure of tactile memory capacity.

## 5. Conclusions

Overall, the present findings provide empirically grounded estimates of performance limits for vibrotactile sequence recall on the forearm under controlled experimental conditions. The results highlight the importance of aligning information density and sequencing demands in wearable haptic systems with human perceptual and cognitive constraints. Using an adaptive staircase procedure, stable threshold estimates were obtained, reflecting the combined influence of spatial and spatiotemporal processing demands in tactile working memory.

On average, performance converged at approximately four items when recall required reproduction of temporal order and at approximately five items when recall depended only on spatial location. This difference indicates that maintaining temporal order imposes an additional constraint on performance compared with spatial recall alone. Although these results should not be interpreted as a universal measure of tactile working memory capacity, they provide useful insight into how sequence length and recall structure influence performance in forearm-based vibrotactile interfaces. Accordingly, the findings offer practical guidance for the design of vibrotactile communication systems, wearable feedback devices, and sensory substitution technologies.

## Figures and Tables

**Table 1:** Characteristics of the Lofelt L5 actuator [34].

Performance Characteristic	Technical Specification
Operating voltage (V)	0–3.3
Frequency range (Hz)	0–15,000
Force (G-p.p.)	0–15
Rising time (ms)	5–15
Operation current (mA)	200–500+

## Data Availability

The data presented in this study are available on request from the Corresponding author. The data is not publicly available due to privacy and project restrictions. Further information about the research and related resources can be found at https://acute.hi.is (accessed on 5 April 2026).
